# Basis Set Limit CCSD(T) Energies for Extended Molecules via a Reduced-Cost Explicitly Correlated Approach

**DOI:** 10.1021/acs.jctc.2c01031

**Published:** 2022-12-28

**Authors:** Mihály Kállay, Réka A. Horváth, László Gyevi-Nagy, Péter R. Nagy

**Affiliations:** † Department of Physical Chemistry and Materials Science, Faculty of Chemical Technology and Biotechnology, Budapest University of Technology and Economics, Műegyetem rkp. 3., H-1111 Budapest, Hungary; ‡ ELKH-BME Quantum Chemistry Research Group, Műegyetem rkp. 3., H-1111 Budapest, Hungary; ¶ MTA-BME Lendület Quantum Chemistry Research Group, Műegyetem rkp. 3., H-1111 Budapest, Hungary

## Abstract

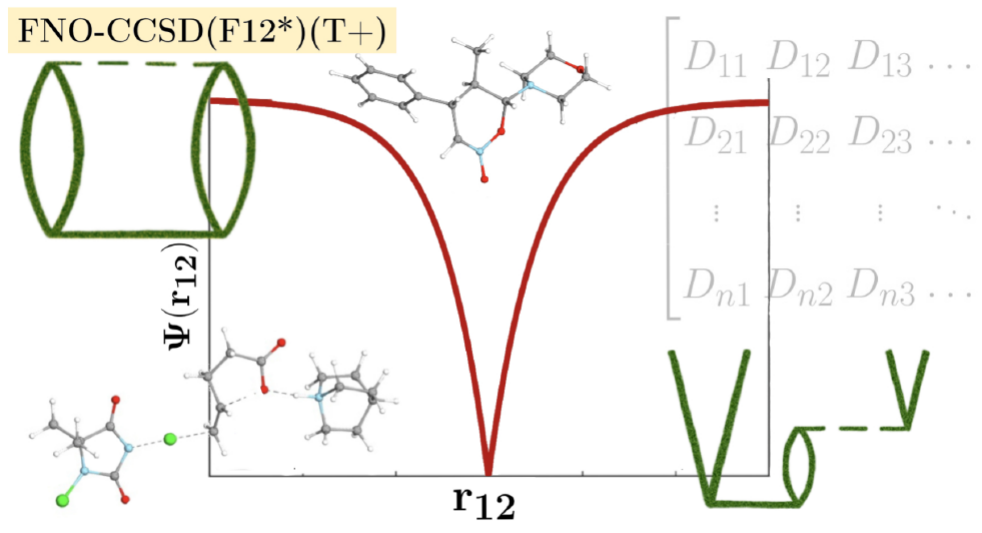

Several approximations are introduced and tested to reduce the computational expenses of the explicitly correlated coupled-cluster singles and doubles with perturbative triples [CCSD(T)] method for both closed and open-shell species. First, the well-established frozen natural orbital (FNO) technique is adapted to explicitly correlated CC approaches. Second, our natural auxiliary function (NAF) scheme is employed to reduce the size of the auxiliary basis required for the density fitting approximation regularly used in explicitly correlated calculations. Third, a new approach, termed the natural auxiliary basis (NAB) approximation, is proposed to decrease the size of the auxiliary basis needed for the expansion of the explicitly correlated geminals. The performance of the above approximations and that of the combined FNO-NAF-NAB approach are tested for atomization and reaction energies. Our results show that overall speedups of 7-, 5-, and 3-times can be achieved with double-, triple-, and quadruple-ζ basis sets, respectively, without any loss in accuracy. The new method can provide, e.g., reaction energies and barrier heights well within chemical accuracy for molecules with more than 40 atoms within a few days using a few dozen processor cores, and calculations with 50+ atoms are still feasible. These routinely affordable computations considerably extend the reach of explicitly correlated CCSD(T).

## 1 Introduction

The reliability of quantum chemical methods strongly depends on what level the electron correlation is taken into account. Even methods that include up to double excitations, such as the coupled-cluster (CC) approach with single and double excitations (CCSD),^1^ are often not sufficient to achieve chemical accuracy (∼1 kcal/mol). There is a growing consensus that the CCSD with perturbative triples [CCSD(T)] approach^2^ is the lowest level method that can provide this accuracy, at least for molecules of single-reference electronic structure. Unfortunately, calculations with CCSD(T) are rather time-consuming since the solution of the CCSD equations scale as , where  is a measure of the system size, while the computation time required for the evaluation of the perturbative triples correction scales as . Furthermore, to achieve the above accuracy goal, rather large one-electron basis sets are required, which also significantly increases the expenses of CCSD(T) calculations as it scales as the fourth power of the basis set size.

There are several approaches in the literature developed to alleviate the aforementioned problems. In the first class of methods, CC calculations are sped up by reducing the size of the molecular orbital (MO) space in which the equations are solved or the perturbative corrections are evaluated. The common feature of these approaches is that the MO basis is divided into an active and an inactive subspace, and the CC calculation is carried out within the former subspace. Prior to that, some transformation is performed in the MO space to maximize the accuracy of the energy evaluated in the truncated space. In the optimized virtual orbitals approaches, a functional depending on the orbital rotation parameters is constructed, and its extremum is determined.^3−7^ A more frequently used approach is the frozen natural orbital (FNO) approximation.^8−10^ Here, a one-particle density matrix is evaluated utilizing a lower-level, usually the first-order Møller–Plesset (MP), wave function.^5,11−13^ The density matrix is diagonalized, and the resulting eigenvectors and eigenvalues are referred to as the natural orbitals (NOs) and the corresponding occupation numbers. The weakly populated NOs are dropped, and the active space is composed of the NOs of larger occupation numbers. The error introduced by this approximation can be efficiently reduced by computing the so-called ΔMP2 correction, which is the difference of the second-order MP (MP2) energies evaluated in the full MO basis and the active space.^3,11^ In addition, the FNO approximation can also be improved by more advanced correction schemes^14^ and by extrapolation techniques.^13,15−18^ The FNO approach was also extended to open-shell systems,^13,19^ higher-order CC methods,^20^ and excited states.^13,19,21−23^ Concerning larger systems, the use of FNO techniques was enabled by reduced-scaling density matrix construction algorithms.^24−27^

A completely different philosophy prevails in explicitly correlated CC methods.^28−30^ Here, the computation time of CC calculations is shortened by reducing their atomic orbital (AO) basis set requirements. This is achieved by adding special configurations to the wave function expansion that explicitly contain the interelectronic distances. The first realization of the explicitly correlated CCSD method was already published in the early 1990s,^31^ but it took a long time for this method to become a viable alternative to the conventional CCSD approach. Thanks to the subsequent improvements in the explicitly correlated infrastructure, such as the introduction of the Slater-type geminal correlation factors (F12),^32^ the complementary auxiliary basis (CABS) approach,^33^ and the efficient explicitly correlated MP2 methods,^34−37^ more competitive explicitly correlated CCSD methods could be developed. The first CCSD model employing the F12 correlation factor (CCSD-F12) was still too expensive for routine applications,^38−40^ but parallel to that, a more approximate explicitly correlated CCSD variant, CCSD(F12), was also introduced,^41,42^ which is 3- to 5- times more expensive than conventional CCSD. The breakthrough came with the development of even more efficient approximations, such as CCSD-F12a and CCSD-F12b,^43,44^,^45,46^ and the CCSD(F12*)^47^ methods. These models are only reasonably more costly than standard CCSD, while preserving the accuracy of the full CCSD-F12 approach.^48^ Explicitly correlated CCSD methods can also be augmented with perturbative triples corrections.^44,49^ For this purpose, probably our (T+) correction published recently is the most appropriate choice^50^ (see Sect. 2.5).

In addition to the FNO and F12 techniques, several other approaches are available to speed up CC calculations. Many modern CCSD(T) implementations, both conventional and explicitly correlated, use the density fitting (DF) approximation.^18,50,51^ Further performance improvement can be attained by data compression techniques, such as the tensor hypercontraction scheme^52−54^ or our natural auxiliary function (NAF) approach.^55^ The DF-CCSD(T) method was also combined with the FNO technique,^56^ and recently we have demonstrated that a combined FNO-NAF-DF approach can result in speedups of 1 order of magnitude for conventional CCSD(T) calculations.^18^ This approach in conjunction with our efficient, parallel, integral-direct CCSD(T) algorithm^57^ significantly broadens the scope of near basis set limit CCSD(T) computations.^18^

A separate class of reduced-cost CCSD(T) methods is formed by the various local CCSD(T) approaches, which utilize the short-term nature of the electron correlation.^58−66^ The common feature of these schemes is that the occupied MOs are localized, and local domains of AOs, virtual orbitals, or fitting functions are assembled for each localized MO or for each pair thereof. These domains are then employed to eliminate the negligible wave function parameters and integrals. The most successful local CC methods also introduce FNO-like approximations and make use of pair- and orbital-specific NOs to further compress the MO space,^67−72^ and these approaches were also combined with F12 techniques to accelerate the basis set convergence of local CCSD(T) calculations.^73−76^ The scaling of CCSD(T) can even be reduced to linear with the aforementioned local correlation approximations,^67−71,77^ but of course, one also has to ensure a sufficient level of convergence for a larger number of such local approximations. Another point to consider is that local approximations start to fully operate for relatively large molecules—typically for systems with 30–50 or more atoms depending on the structure. Under this range, optimized CCSD(T) implementations or CCSD(T) approaches utilizing FNO and related approximations are competitive, as we have recently demonstrated for conventional CCSD(T).^18^

In this study, our intention is to develop a reduced-cost explicitly correlated CCSD(T) approach that is as accurate as the parent method and more efficient than local CCSD(T) approximations for molecules of a couple of dozens of atoms. Motivated by the success of the combined FNO-NAF approach for conventional CCSD(T),^18^ we embark on adapting these approximations to explicitly correlated CCSD(T) methods. Furthermore, we also inspect the possibilities for the reduction of the size of the CABS—a concept that does not occur for conventional CCSD(T). We also discuss the required modifications for the (T+) correction when the latter is used together with explicitly correlated CCSD models. Finally, we demonstrate that the new FNO-CCSD(F12*)(T+) method can approach the basis set limit for up to 53-atom molecules using only a few dozen compute cores within a few days of wall time.

## 2 Theory

First, to facilitate the understanding of the following discussion, we briefly summarize the special features of explicitly correlated CCSD methods. Then, the various approximations are introduced to reduce their computational costs. Finally, we consider the necessary modifications in the evaluation of the perturbative triples correction when the latter is employed together with the reduced-cost CCSD-F12 approximations developed.

### 2.1 Explicitly Correlated CCSD Methods

In conventional CCSD theory,^1^ the wave function is written in an exponential form as
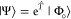
1where  is the reference determinant and  denotes the cluster operator with
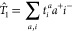
2and
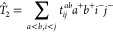
3Here,  and  are the cluster amplitudes, and indices *i*, *j*, ... (*a*, *b*, ...) refer to occupied (virtual) spin orbitals, while *p*, *q*, ... will label generic MO indices. Operators *a*^+^ and *i*^–^ are creation and annihilation operators, respectively. The CCSD energy, *E*^CCSD^, and the wave function parameters are obtained by substituting eq 1 into the Schrödinger equation and projecting onto the space of the reference and the singly  and doubly  excited determinants as
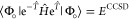
4
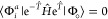
5
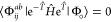
6where  is the Hamiltonian.

In explicitly correlated approaches,^28−30^ the wave function is augmented with geminals
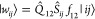
7explicitly containing the interelectronic distances *r*_12_. Here,  is the F12 correlation factor with γ as an exponent, and  stands for a strong orthogonality projector, which orthogonalizes the pair functions to all possible products of the Hartree–Fock (HF) MOs. For the latter, almost all modern F12 approaches use ansatz 2,
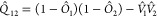
8or one of its approximate forms, where  and  are the projectors onto the space of occupied and virtual HF MOs, respectively, and the subscript refers to the number of the electron. Operator  is defined by the
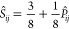
9expression, where  permutes the spatial components of spin orbitals *i* and *j* in determinant |*ij*⟩. In practice, the functions |*w*_*ij*_⟩ are represented by an expansion in determinant basis |*αβ*⟩ formed from a formally complete virtual basis, hereafter indexed by α, β, .... In the CABS approach,^33^ which is also utilized in this study, this virtual basis is formed from the HF virtual MOs and a complementary MO basis. To construct the latter, an AO-like auxiliary basis is employed. The functions of this basis are orthogonalized to the HF MOs, and the resulting orbitals are canonicalized, that is, the Fock-operator is diagonalized in their basis. Hereafter, *a*′ and *b*′ will stand for the CABS representation of the complementary virtual orbitals, while the orbitals in the HF MO plus CABS basis will be denoted by *p*′ and *q*′. With the aid of the complete virtual basis, the explicitly correlated geminals can be represented by the
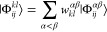
10combination of determinants, where .

In explicitly correlated CCSD theories,^38,40,42,43,46,47^ the cluster operator also incorporates an additional operator,
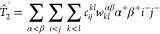
11generating double excitations into the above pair functions with  as the corresponding amplitudes. Equations 4–6 still hold in the explicitly correlated case with the modified cluster operator , and further equations,
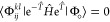
12are needed to determine the  coefficients. However, in practice, eqs 4–6 and 12 are significantly simplified in the state-of-the-art explicitly correlated CCSD approaches. In addition to the extensive use of the resolution of identity (RI) approximation for the many-electron integrals, small higher-order  contributions are neglected. The method can further be improved by the fixed-amplitude approach.^78^ Here, the coefficients  are fixed at , which not only guarantees the fulfillment of the cusp conditions for singlet and triplet electron pairs but also enhances computational efficiency. In the most advanced explicitly correlated CCSD methods,^43,47^ if the fixed-amplitude approximation is invoked, all  contributions can be computed once before solving the CCSD equations. These F12-dependent contributions can be absorbed in the conventional CCSD intermediates, which also means that the operation count for the solution of the CCSD equations is hardly affected, and there is no reference to the complementary basis. The evaluation of the F12-dependent terms scales as , where *n*_o_, *n*_v_, and *n*_c_ are the number occupied, virtual, and complementary virtual orbitals, respectively, whereas the scaling of the CCSD equations still does not exceed .

From our point of view, it is important to note that the latter explicitly correlated CCSD methods also require the calculation of the MP2-F12 correlation energy in advance of the solution of the CCSD equations. The uncoupled contributions in the explicitly correlated part of the MP2-F12 correlation energy are also added to the CCSD correlation energy. These are the pure contributions of the explicitly correlated geminals, excluding the coupling to . The latter is the contribution of intermediate *C* of MP2-F12 theory to the MP2-F12 correlation energy [see, e.g., eqs 7 and A3 of ref (50)]. The missing coupling contributions are evaluated from the CCSD amplitudes. In fact, the major contribution to the explicitly correlated part of the CCSD correlation energy comes from MP2-F12.

It is also important to note that explicitly correlated MP2 and CCSD implementations intensively make use of the DF approximation. In this approach, an auxiliary fitting basis is used, whose elements will be labeled by indices *P*, *Q*, .... The four-center integrals are approximated from two- and three-center ones including the fitting functions. Four-center Coulomb integrals, (*pq*|*rs*) in the (11|22) convention, can be evaluated as
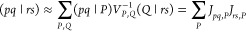
13with
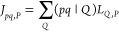
14where *V*_*P*,*Q*_ = (*P*|*Q*) and (*pq*|*P*) are two- and three-center Coulomb integrals, respectively, and  is a shorthand notation for the corresponding element of the inverse of the two-center integral matrix. Matrix **L** is obtained by an appropriate decomposition of matrix **V**^–1^ as **V**^–1^ = **LL**^T^. Most frequently, **L** is defined by **V**^–1/2^, but a better strategy is to use the Cholesky-decomposition of **V**^–1^. Unfortunately, for the other types of integrals appearing in explicitly correlated theories, that is, the integrals of operators *f*_12_, , *f*_12_/*r*_12_, and  with  as the del operator with respect to the coordinates of the first electron, robust fitting formulas must be used.^37,79,80^ For instance, the integrals of *f*_12_ are evaluated as
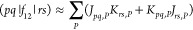
15where
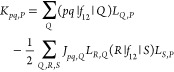
16and similar equations hold for the three other operators.

### 2.2 Frozen Natural Orbitals

In conventional FNO-CCSD(T) calculations, the virtual–virtual block of the MP2 one-particle density matrix,
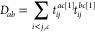
17is constructed, where  is an amplitude of the first-order MP wave function. This matrix is diagonalized, and of the resulting eigenvectors, i.e., the NOs, those are retained where the corresponding eigenvalue, i.e., the occupation number, is greater than a threshold. The selected NOs are canonicalized, which is not mandatory for CCSD but is necessary for the perturbative triples correction so that the HF-based expressions can be used. The CCSD correlation energy lost is usually approximated by the ΔMP2 correction, which is the difference of MP2 energies in the full and the truncated basis. Please note that the eigenvectors of the density matrix are equivalent to the right singular vectors of  if it is regarded as a matrix with the composite row index *aij* and column index *b*.^81^ Thus, the FNO approach is practically a singular value decomposition (SVD) of the first-order wave function generating a truncated basis in which the wave function is best approximated. In other words, this procedure is a SVD-based rank reduction for the amplitude matrix.

The adaptation of this approach to explicitly correlated CC theory is not entirely trivial. In principle, one could construct a one-particle density matrix for MP2-F12, but this would be rather complicated and relatively costly. Thus, the most plausible choice is to use the conventional MP2 NOs as defined above. However, a number of further considerations are warranted.

First, the FNO approximation can be applied not only at the CC level but also in the initial MP2-F12 calculation. In this way, the above FNOs could speed up the MP2-F12 calculation at the expense of some loss in the correlation energy. Considering that the evaluation of the MP2-F12 correlation energy is relatively cheap with respect to the subsequent CC calculation and a significant part of the explicitly correlated contribution to the CC energy stems from MP2-F12, we refrain from employing the FNO approach at the MP2-F12 level. A separate study will be dedicated to the cost-reduction of standalone MP2-F12 using the techniques elaborated herein.

Second, it is in question how the strong orthogonality projector of eq 8 should be defined in the FNO-based approach, that is, if operators  and  should project onto the truncated or the original virtual HF MO space. Since the main purpose of  is to keep the explicitly correlated pair functions orthogonal to all conventional configurations, if the MO space and thereby the space of the latter configurations is truncated, it is more consistent to also redefine  and use the truncated virtual space at the construction of the projectors. Furthermore, the second option would result in a significantly more complicated algorithm and the loss of the advantages of the FNO approximation at the evaluation of the F12-dependent intermediates. Consequently, we go with the first option. This choice is also supported by the experience gained for local correlation methods, where it was found that it is more advantageous to define  for an orbital pair in the corresponding truncated domain of virtual MOs rather than in the entire virtual space.^82,83^

Third, we should keep in mind that the complementary basis is a virtual MO basis that is orthogonal to the original HF MOs. Thus, if the NOs of small occupation number are dropped, there will be a gap between the space of the correlated virtual MOs and the complementary virtual space. This may result in considerable errors since the basis used for the RI approximations is not optimal. Therefore, we add the dropped NOs to the complementary space. Note that the new complementary virtual space does not need to be canonicalized because it is only used at the computation of CC intermediates, and these are invariant to the unitary transformation within that space.

Fourth, the contribution of the dropped NOs to the CCSD correlation energy is missing just as in the case of conventional CCSD. It is compensated neither by the MP2-F12 correlation energy contribution to the CCSD energy nor by that the dropped NOs are added to the complementary basis. Thus, it is justified to include the ΔMP2 correction even in the explicitly correlated case.

Fifth, as pointed out in Section 2.1, the coupling terms of the explicitly correlated and the conventional configurations occur in the CCSD energy and residual equations. However, the contributions of those conventional excitations where the excitation takes place to a dropped virtual NO are lacking. Since this may also cause significant error, we calculate the missing contribution at the MP2 level. In practice, the whole coupling contribution is evaluated first with the original MO and CABS bases and then with the truncated and canonicalized NO basis using the extended complementary basis. The difference of the two is also added to the CCSD correlation energy in addition to the ΔMP2 correction.

With the above modifications, the FNO approach can be used for explicitly correlated CCSD calculations as for conventional CCSD. We note that FNOs could also be used to speed up standalone MP2-F12 calculations. Since the primary focus of this study is the cost reduction of explicitly correlated CC calculations, and otherwise the applicability of MP2-F12 is limited, we do not discuss this aspect in detail in the present study.

### 2.3 Natural Auxiliary Functions

In our NAF approach,^55^ motivated by the SVD formulation of the FNO approximation, the right singular vectors of matrix **J** defined by eq 14 are determined supposing that *pq* is a composite row index. In practice, the symmetric matrix
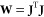
18is diagonalized. The eigenvectors with small eigenvalues, that is, the singular vectors with small singular values, are dropped, and the auxiliary index of **J** is transformed to the truncated basis. In this way, **J** is represented in a new, molecule-specific fitting basis of lower dimension, the NAF basis, which guarantees that the compressed matrix is the best approximation to the original one. The NAF technique significantly lowers the costs of the transformation and processing of three-center integrals and the assembly of the four-center integrals (eq 13). As we have recently demonstrated, it is quite efficient in speeding up both conventional^18,21^ and various local approximation based correlated calculations,^84−87^ especially if it is combined with the FNO approach.

If we intend to generalize the NAF technique to explicitly correlated calculations, we should keep in mind that not only Coulomb integrals enter the calculation but four other types appear as well. Theoretically, one could define separate NAF bases for each type of integrals using the corresponding three-center integrals. However, it would require the transformation of matrix **J** to five different fitting bases and the storage of the resulting five lists since the assembly of all types of integrals requires the Coulomb integral **J** [cf. eqs 15 and 16]. Thus, it seems to be a much better strategy to still define the NAFs by diagonalizing matrix **W** and to employ this fitting basis for each integral type.

The NAF technique can potentially be deployed at three points in an explicitly correlated CCSD calculation that uses the DF approach. In principle, it can be used already at the initial MP2-F12 run as well as at the construction of the F12-dependent intermediates and in the CCSD iterations. The application of NAFs would be particularly useful for MP2-F12 calculations because their expenses are dominated by the assembly of the four-center integrals, which operation scales with the size of the fitting basis. However, for the reasons outlined in Section 2.2, we refrain from this here, and we will investigate in a later work how much standalone MP2-F12 calculations can benefit from the use of NAFs.

Considering the other two possibilities, the F12-dependent intermediates and the CCSD iterations, we should realize that for the former, matrix **J** also carries one index of the complementary space, whereas for the latter, **J** depends only on the orbitals of the conventional MO basis. Since the major advantage of the NAF technique is that it constructs a reduced fitting basis optimal for the given MO basis, it is thus recommended to use different NAF bases at the two places. Consequently, for the CCSD iterations, we go with the NAFs derived from *J*_*pq*,*P*_-type integrals and use the infrastructure elaborated for conventional CCSD as described in ref (18). For the F12-dependent intermediates, the NAFs are constructed from *J*_*p*′*q*,*P*_-type matrix elements. In the latter case, after diagonalizing **W**, both **J** and the **K**-type integrals are transformed to the truncated NAF basis, but no further modification is needed in the algorithm for the evaluation of the F12-dependent intermediates. The construction of the NAF basis and the transformation of the integrals to the new fitting basis scale as the fourth power of the basis set size, thus the overhead due to these operations is relatively low with respect to the fifth-power scaling operations, eqs 13 and 15, sped up by this approach.

The application of the NAF technique to open-shell systems requires further considerations. As discussed in ref (55), here, we have separate *J*_*pq*,*P*_ lists for the alpha and beta MOs in the case of conventional CC methods. By default, separate **W** matrices are computed for both, and their average is used to construct the NAFs. Alternatively, the NAF basis can be derived exclusively from the integrals of the alpha MOs, which somewhat decreases the overhead of the NAF construction. For explicitly correlated methods, the situation is even more complicated. For the latter, due to the presence of the operators , we also need *J*_*p*′*q*,*P*_ lists where *p*′ is an alpha orbital, and *q* is beta and vice versa. Here, we also have the cheaper option of computing the NAFs using only the *αα*-type list, but by default, the NAFs are determined by averaging the matrices **W** evaluated from the various spin cases, *αα*, *αβ*, *βα*, and *ββ*. The latter choice is preferred because it is expected to generate a NAF basis that is balanced for all four types of lists at the same time.

For the compensation of the error introduced by the use of the NAF technique during the CCSD iteration, the same corrections are useful as in the conventional case.^18^ That is, the MP2 correlation energy is computed with and without the NAF approximation, and the difference of the two is added to the final CCSD correlation energy. Furthermore, at the evaluation of the correlation energies the amplitudes are contracted with integrals computed without the NAF approximation. The processing of the required *J*_*ai*,*P*_-type lists in the original fitting basis and the assembly of the (*ai*|*bj*) integrals from these lists just negligibly increase the computation time. Unfortunately, for the reduction of the error brought in by the application of the NAF approximation at the evaluation of F12-dependent intermediates, no such inexpensive corrections can be calculated.

### 2.4 Natural Auxiliary Basis

In Sections 2.2 and 2.3, we have demonstrated that both the MO and the fitting basis can be reduced by SVDs applied to the appropriate quantities. In explicitly correlated calculations, there is a third type of basis, the CABS. Now the question arises if the dimension of the latter can also be shrunk by similar data compression techniques. Here, we seek the answer to this question.

As the complementary basis is employed for the second-quantized representation of the explicitly correlated geminals [cf. eq 10], from the theoretical point of view, a justifiable solution would be to construct a reduced complementary basis with constraining the coefficients  in the new basis to be as close to their original values as possible. In practice, only  -type matrix elements are computed in an explicitly correlated calculation, which, inserting projector  given by eq 8, reduce to integrals . One can also argue that the primary purpose of the auxiliary basis is to approximate the three- and four-electron integrals entering the explicitly correlated theory; thus, the goodness of the basis can also be measured by the accuracy of the arising two-electron integrals appearing in the CC equations. In addition to the aforementioned integrals of *f*_12_, these include the analogous integrals of *f*_12_/*r*_12_. Thus, in both cases, a pragmatic procedure would be to perform the SVD of the corresponding four-index tensors supposing that they are two-index matrices with composite *bij* row index and *a*′ column index. This is, unfortunately, a fifth-power scaling operation with a relatively large prefactor. For this reason, we opted for an even more pragmatic solution: instead of the four-center integrals, we decompose the three-index quantities of which they are constructed. One expects that, if the three-index integrals are well represented in the new complementary basis, the four-center integrals computed thereof will also be accurate. Here, there are still two options: we can decompose either integrals *J*_*a*′*i*,*P*_ or *K*_*a*′*i*,*P*_. Although the latter choice seems to be more satisfactory from the theoretical point of view because these are the three-index integrals of the *f*_12_ correlation factor, it is more tempting to take integrals *J*_*a*′*i*,*P*_ as these quantities are also required for the evaluation of all other F12-dependent intermediates. In addition, the computation of these intermediates also needs the *J*_*a*′*p*,*P*_-type integrals, thus we finally use the latter for the definition of the truncated CABS basis. The superiority of the integrals *J*_*a*′*p*,*P*_ for this purpose over *J*_*a*′*i*,*P*_ or *K*_*a*′*i*,*P*_ was also verified by numerical experiments.

In practice, similar to the FNOs or NAFs, the SVD is carried out by building and diagonalizing matrix  with elements
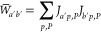
19The eigenvectors of  define a new complementary basis with the corresponding eigenvalues as the populations. The orbitals of low population are dropped, and the resulting truncated complementary basis, which we call the natural auxiliary basis (NAB), is used thereafter. Integrals *J*_*a*′*p*,*P*_, *K*_*a*′*p*,*P*_, and the analogous integrals of the other operators are transformed to the NAB, and then the algorithms for the evaluation of the F12-dependent intermediates are executed without any modification.

Though the overhead of the construction of the NAB is rather low, the evaluation of the intermediates requiring the *J*_*a*′*p*,*P*_ or similar types of integrals takes a relatively small fraction of the entire computation time as well. Consequently, only moderate speedups can be expected for explicitly correlated CCSD approaches that treat the F12-dependent intermediates noniteratively. The NAB technique can be particularly effective for the models that evaluate those terms iteratively. Moreover, we expect considerable gain for MP2-F12 calculations, where the operation count for most of the terms depends on the size of the complementary basis. However, for the reasons discussed above, we will consider the cost reduction of MP2-F12 in a forthcoming study. We also mention that the NAB approach is potentially well suited for explicitly correlated local CC implementations, where the processing of three-center integrals is one of the bottlenecks.

It is also pertinent to comment on the joint use of the FNO, NAF, and NAB approximations. In principle, any two of the three or all three can be employed at the same time. If the FNO and NAB approaches are applied together, it is recommended to construct the FNOs before the NAB. In this way, the complementary basis extended by the dropped NOs enters the NAB construction algorithm, and a NAB optimal for the final FNO basis is generated. If the NAFs are also used, they can also be constructed at any point. Nevertheless, it is most advantageous to calculate them at the end, after constructing the FNOs and the NAB. As pointed out in Section 2.3, the NAF basis is a fitting basis tailored to the given MOs, hence, it is recommended to determine it when the MO bases reached their final form.

Finally, to help the reader, the various approximations considered in our study are summarized in Table 1. In the second column of the table, the basis is given the dimension of which is reduced by the approximation via the SVD of the quantity specified in column 3. From the rightmost column, it can be inferred whether the approximation functions at the evaluation of F12-dependent terms or during the CC iterations and computation of the perturbative triples correction. Notice that, if the NAF and NAB approaches are employed together with the FNO approximation, the *p* and *q* indices of integrals *J*_*p*′*q*,*P*_, *J*_*pq*,*P*_, and *J*_*a*′*p*,*P*_ run over the truncated NO basis.

**Table 1:** Summary of the Various Approximations Used to Speed up Explicitly Correlated CCSD(T) Calculations

Approximation	Truncated basis	SVD^a^	Application^b^
FNO	virtual HF MO (*a*)	*t*_*ij*_^*ab*[1]^	F12, CC
NAF	DF auxiliary (*P*)	*J*_*p*′*q*,*P*_	F12
		*J*_*pq*,*P*_	CC
NAB	complementary MO (*a*′)	*J*_*a*′*p*,*P*_	F12

a The quantity the SVD of which is used to construct the truncated basis.

b The approximation is applied to reduce the corresponding dimension of F12-dependent intermediates (F12) or integrals, intermediates, and cluster amplitudes entering the CCSD(T) equations (CC).

### 2.5 Perturbative Triples Correction

The treatment of triple excitations in explicitly correlated CC methods is not straightforward. The simplest solution is to calculate the (T) correction with the converged explicitly correlated CCSD amplitudes. If this route is followed, the FNO and NAF approaches can also be used to speed up the evaluation of the (T) correction as described in ref (18) for conventional CCSD(T).

On the other hand, recently we have proposed a more advanced perturbative triples correction, termed (T+), which reduces the basis set error of (T).^50^ The basic idea was to split up the MP2 and MP2-F12 correlation energies and the triples correction into the contribution of occupied MOs, respectively, as
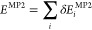
20
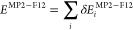
21and
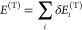
22Explicit expressions for the , , and  increments can be found in ref (50). Supposing that the contributions of a particular MO to the MP2 correlation energy and the (T) correction scale similarly with the basis set size, we can scale the contribution of each MO to the (T) correction separately with the ratio of the corresponding  and  increments as
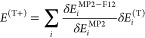
23

If the *E*^(T+)^ correction is evaluated with the FNO approach, the important difference is that the  contributions are computed in the reduced semicanonical NO space, while the  increments are not affected. Consequently, it is recommended to evaluate the  contributions in the reduced space as well. Otherwise, the calculation of the *E*^(T+)^ correction can be carried out without any further modification.

## 3 Benchmark Calculations

### 3.1 Computational Details

The cost-reduction techniques discussed herein have been implemented in the Mrcc quantum chemistry suite,^88,89^ which was also used in all the calculations. Our explicitly correlated CCSD(T) implementation was described in ref (50), while the development of the underlying hand-optimized, parallel, and (partially) integral-direct conventional DF-CCSD(T) program was reported in refs (57), (18), and (89).

For the explicitly correlated CCSD calculations, the CCSD(F12*) model of Hättig and co-workers was employed^47^ in conjunction with our (T+) correction for the triple excitations.^50^ Restricted open-shell HF references were used for the open-shell systems. The frozen core approximation was applied in all correlation calculations.

The correlation consistent *X*-tuple-ζ cc-pV*X*Z-F12 (*X* = D, T, Q) AO basis sets developed for explicitly correlated calculations^90^ and the corresponding cc-pV*X*Z-F12-OPTRI CABS bases were employed.^91,92^ For the sake of brevity, the cc-pVDZ-F12, cc-pVTZ-F12, and cc-pVQZ-F12 basis sets will be referred to as DZ, TZ, and QZ, respectively. The DF approximation was used throughout for both the HF and the correlation calculations with the aug-cc-pV(*X*+1)Z-RI-JK^93^ and the aug-cc-pwCV(*X*+1)Z-RI^94^ fitting bases, respectively. Slater-type *f*_12_ correlation factors with exponents of 0.9, 1.0, and 1.1 were applied with the cc-pVDZ-F12, cc-pVTZ-F12, and cc-pVQZ-F12 basis sets, respectively.^90^ To facilitate the evaluation of the corresponding integrals, the correlation factors were approximated by linear combinations of six Gaussians.^95^

In our benchmark calculations, the test set of Knizia, Adler, and Werner (KAW)^44^ was used. This set includes 49, 28, and 48 atomization energies and reaction energies of closed- and open-shell systems, respectively, involving 66 species. The reference CCSD(F12*)(T+) and complete basis set (CBS) limit CCSD(T) energies were taken from ref (50). Here, we only present the overall error measures for the 125 thermochemical processes in terms of the corresponding mean absolute errors (MAEs), root-mean-square (RMS) deviations, and maximum errors (MAXs). The separate error statistics for the atomization and closed- and open-shell reaction energies can be found in the Supporting Information (SI), where the computed correlation energies, thermochemical quantities, errors of correlation energies, and the percentage of the retained orbitals (auxiliary functions) are also presented. Since the errors of the reduced-cost CCSD(F12*)(T+) approaches with respect to the CBS CCSD(T) reference values will be compared with the corresponding errors of the parent CCSD(F12*)(T+) method, the latter are displayed in Table 2.

**Table 2:** Errors (in kJ/mol) of CCSD(F12*)(T+) Energy Differences with Respect to CBS CCSD(T) Reference Values for the KAW Test Set

	Basis set		
Error measure	cc-pVDZ-F12	cc-pVTZ-F12	cc-pVQZ-F12
MAE	3.5	1.1	0.5
RMS	4.5	1.4	0.6
MAX	19.0	3.9	2.0

Further benchmark calculations were carried out for the test set developed by Adler and Werner (AW),^96^ which incorporates 52 reactions of 58 closed-shell molecules of up to 18 atoms. Here, the reference CCSD(F12*)(T+)/cc-pV*X*Z-F12 reaction energies computed in this study are employed. The wall-clock time measurements for the AW set were carried out on an 8-core Intel Xeon E5-2609 v4 processor running at 1.7 GHz. The larger computations of Section 3.7 were performed with 28-core Intel Xeon Platinum 8180M CPUs clocked at 1.7 GHz.

### 3.2 Performance of the FNO Approximation

First, we discuss the performance of the FNO approach for energy differences. The relevant error measures for the KAW test set are compiled in Table 3. Here, just as in the following tables, we present the errors with respect to both the parent CCSD(F12*)(T+) energy differences evaluated with the same basis set and the corresponding CBS-limit CCSD(T) values. The former statistical measures directly quantify the error introduced by the cost-reduction techniques, whereas the latter characterize the overall error brought in by the finite basis set used in the explicitly correlated approach and our cost-reduction approximations. Since the main purpose of the explicitly correlated methodology is to approach the CBS-limit as close as possible, the latter error measures are more informative concerning the applicability of the cost-reduction techniques developed.

**Table 3:** Errors (in kJ/mol) of Approximate CCSD(F12*)(T+) Energy Differences Using the FNO Approach with Various Thresholds with Respect to CCSD(F12*)(T+) and CBS CCSD(T) (in Parentheses) Reference Values for the KAW Test Set

		Threshold				
Basis set	Error	10^–4^	5 × 10^–5^	10^–5^	5 × 10^–6^	10^–6^
cc-pVDZ-F12	MAE	1.4 (3.3)	1.5 (3.0)	0.7 (3.3)	0.4 (3.4)	0.3 (3.6)
	RMS	2.2 (4.2)	2.3 (4.1)	1.0 (4.4)	0.7 (4.5)	0.7 (4.6)
	MAX	7.7(16.8)	7.8(19.2)	3.2(21.3)	2.2(21.2)	4.5(19.0)
cc-pVTZ-F12	MAE	1.3 (1.5)	1.5 (1.5)	1.0 (1.0)	0.6 (1.0)	0.3 (0.9)
	RMS	1.9 (2.2)	2.2 (2.0)	1.4 (1.4)	1.0 (1.3)	0.5 (1.2)
	MAX	10.1(12.7)	6.6 (6.0)	4.4 (4.8)	3.5 (4.0)	1.8 (3.9)
cc-pVQZ-F12	MAE	1.3 (1.4)	1.4 (1.4)	0.9 (1.0)	0.6 (0.7)	0.2 (0.4)
	RMS	1.9 (2.0)	2.1 (2.0)	1.4 (1.3)	1.0 (1.0)	0.3 (0.6)
	MAX	6.8 (7.5)	6.4 (6.5)	4.6 (4.6)	3.2 (3.9)	1.9 (2.0)

If the errors with respect to the parent CCSD(F12*)(T+) method are considered, the average errors are quite basis set independent, while the scatter of maximum errors is more arbitrary. The convergence with the truncation threshold is monotonic from 5 × 10^–5^, excepting the MAX for the DZ basis. If a MAE of 1 kJ/mol is tolerated, the threshold of 10^–5^ is sufficient with all basis sets in accordance with our experience for conventional CCSD(T).^18^ If the MAX is required to be lower than 1 kcal/mol, a threshold of 5 × 10^–6^ is needed for the two larger basis sets. If the various thermochemical properties are considered separately (see SI), as expected, the error of the atomization energies is larger than that for the reaction energies. For the latter, the MAXs are safely below 1 kcal/mol in any case. It is also instructive to inspect the sources of the errors. If we look at the relative errors of the correlation energies (see SI), we find that larger inaccuracies can be observed for atoms and systems containing second-row elements. Accordingly, for processes involving such species, larger errors are expected, especially when the number of free atoms is different on the two sides of the chemical equation.

The picture is somewhat more nuanced when the errors of the FNO-based CCSD(F12*)(T+) approach with respect to the CBS-limit CCSD(T) references (numbers in parentheses in Table 3) are compared to the intrinsic errors of CCSD(F12*)(T+) (see Table 2). With the DZ basis set, the errors hardly change with any truncation threshold considered. In fact, the error measures are lower in most cases. This primarily comes from the strong error cancellation for the atomization energies, but the errors for the reactions energies are still favorable (see SI). This means that all the thresholds can be used, even the least rigorous one, 10^–4^. For the other two bases, the results slowly deteriorate when loosening the truncation parameter. With the TZ basis, 10^–5^ seems to be a good choice since the MAE and RMS are practically not affected, only the MAX worsens by 0.9 kJ/mol. For the QZ basis set, even with the tight threshold of 5 × 10^–6^, the error measures, in particular MAX, are close to those obtained with the TZ basis. Thus, to reserve the accuracy of the QZ basis, 10^–6^ should be chosen.

All in all, we recommend the thresholds of 10^–4^, 10^–5^, and 10^–6^ for the DZ, TZ, and QZ bases, respectively. With these parameters, in average, 62, 77, and 86% of the virtual NOs are retained, respectively (see SI). Taking into account that the computation time of a CCSD(F12*)(T+) calculations scales as the fourth power of *n*_v_, respective speedup factors of up to 7, 3, and 2 are anticipated.

### 3.3 Performance of the NAF Approximation

As explained in Section 2.3, the NAF approximation can be used at two points in an explicitly correlated CC calculation: at the computation of the F12-dependent intermediates and in other parts of the CC calculation where just the normal Coulomb integrals are utilized. Since the NAF approach presumably behaves differently in the two situations, they are discussed separately.

In Table 4, the error statistics are presented for the case when the NAF approach is only employed for F12-dependent intermediates. Notice that these are constructed only once prior to the CC iterations in the case of the CCSD(F12*)(T+) model. Hence, the inaccuracy caused by the use of NAFs is expected to be moderate, but in turn, less gain in the computation time is foreseen compared to the FNO approach.

**Table 4:** Errors (in kJ/mol) of Approximate CCSD(F12*)(T+) Energy Differences Using the NAF Approach at the Construction of F12-Dependent Intermediates with Various Thresholds (in a.u.) with Respect to CCSD(F12*)(T+) and CBS CCSD(T) (in Parentheses) Reference Values for the KAW Test Set

		Threshold				
Basis set	Error	2 × 10^–1^	10^–1^	7.5 × 10^–2^	5 × 10^–2^	10^–2^
cc-pVDZ-F12	MAE	0.9 (3.9)	0.2 (3.5)	0.1 (3.5)	0.1 (3.5)	0.0 (3.5)
	RMS	1.1 (5.1)	0.3 (4.6)	0.2 (4.5)	0.1 (4.5)	0.0 (4.5)
	MAX	2.9(18.5)	0.8(18.3)	0.6(18.6)	0.3(19.1)	0.0(19.0)
cc-pVTZ-F12	MAE	0.3 (1.3)	0.1 (1.1)	0.1 (1.1)	0.0 (1.1)	0.0 (1.1)
	RMS	0.4 (1.6)	0.1 (1.4)	0.1 (1.4)	0.0 (1.4)	0.0 (1.4)
	MAX	1.5 (5.2)	0.4 (4.1)	0.3 (4.1)	0.1 (3.9)	0.0 (3.9)
cc-pVQZ-F12	MAE	0.2 (0.6)	0.0 (0.5)	0.0 (0.5)	0.0 (0.5)	0.0 (0.5)
	RMS	0.3 (0.8)	0.0 (0.7)	0.0 (0.7)	0.0 (0.6)	0.0 (0.6)
	MAX	1.6 (3.1)	0.2 (1.8)	0.1 (1.9)	0.0 (2.0)	0.0 (2.0)

If the errors with respect to the parent CCSD(F12*)(T+) method are considered, we realize that the convergence with the truncation threshold is monotonic. The errors are only moderately basis set dependent and practically disappear with the threshold of 10^–2^ a.u. The average (maximum) errors are not larger than 1 kJ/mol (1 kcal/mol) using 2 × 10^–1^ a.u. and tighter thresholds with any basis set. Again, the inaccuracy is considerably larger for the atomization energies than that for the reaction energies (see SI), and it is also true that the performance of the approximation is weaker for species containing second-row elements. If the errors relative to the CBS-limit are scrutinized (parenthesized numbers in Table 4), we can conclude that the errors are practically not affected with thresholds of 10^–1^ a.u. and smaller. On the whole, we propose a default threshold of 10^–1^ a.u. for all three basis sets. With this parameter, on the average, 56, 61, and 68% of the NAFs are retained, respectively, with the DZ, TZ, and QZ basis sets (see SI).

If the NAF approach is employed for the construction of Coulomb integrals in the CC calculation (see Table 5), the inaccuracy of the computed energy differences is higher as the iterations amplify the errors, and this approximation directly affects the total correlation energy, not only the F12 contribution. The basis set dependence of the errors is also more pronounced. The errors with respect to the original CCSD(F12*)(T+) energies converge monotonically and vanish for thresholds of lower than 5 × 10^–3^ a.u. The MAEs are lower than 1 kJ/mol with the truncation parameter 5 × 10^–2^ a.u., and the MAX is only greater than 1 kcal/mol with the DZ basis. The inaccuracy of the atomization energies is still 2- to 3-times larger than that of the reaction energies, and the largest errors can again be observed for the second-row elements (see SI). Turning to the absolute errors relative to the CBS limit, we can state that the 5 × 10^–2^ a.u. threshold still seems to be adequate because with this parameter, the error measures are slightly better due to error cancellation with the TZ and QZ basis sets and just moderately grow with DZ. Consequently, we chose this threshold as default but also note that for the QZ basis, even looser parameters are acceptable. With the default threshold, 28, 35, and, 46% of the NAFs are retained with the DZ, TZ, and QZ bases, respectively. As expected, the Coulomb integrals during the CC calculation can be fitted with a lower number of fitting functions than the F12-dependent intermediates since for the latter, both Coulomb and F12-dependent integrals must be fitted with the same auxiliary basis, and the relevant integrals also include functions in the complementary virtual basis.

**Table 5:** Errors (in kJ/mol) of Approximate CCSD(F12*)(T+) Energy Differences Using the NAF Approach in the CC Calculation with Various Thresholds (in a.u.) with Respect to CCSD(F12*)(T+) and CBS CCSD(T) (in Parentheses) Reference Values for the KAW Test Set

		Threshold				
Basis set	Error	10^–1^	7.5 × 10^–2^	5 × 10^–2^	10^–2^	5 × 10^–3^
cc-pVDZ-F12	MAE	5.3 (7.3)	2.8 (5.5)	0.9 (4.1)	0.1 (3.5)	0.0 (3.5)
	RMS	6.7 (9.6)	4.9 (7.7)	1.2 (5.3)	0.1 (4.5)	0.0 (4.5)
	MAX	20.9(34.3)	22.5(27.3)	5.1(18.7)	0.3(18.8)	0.1(18.9)
cc-pVTZ-F12	MAE	1.3 (1.7)	0.2 (1.0)	0.1 (1.1)	0.0 (1.1)	0.0 (1.1)
	RMS	2.2 (2.7)	0.3 (1.3)	0.2 (1.4)	0.0 (1.4)	0.0 (1.4)
	MAX	10.2(13.5)	1.1 (4.0)	0.9 (3.2)	0.2 (3.9)	0.0 (3.9)
cc-pVQZ-F12	MAE	0.4 (0.7)	0.1 (0.4)	0.1 (0.5)	0.0 (0.5)	0.0 (0.5)
	RMS	0.6 (1.0)	0.2 (0.6)	0.1 (0.6)	0.0 (0.6)	0.0 (0.6)
	MAX	2.8 (3.4)	0.7 (1.9)	0.4 (1.9)	0.0 (1.9)	0.0 (2.0)

### 3.4 Performance of the NAB Approximation

The error metrics for the KAW test set using the NAB approximation are displayed in Table 6. As we can see, the errors are rather basis set dependent. They converge monotonically with the cutoff parameters, apart from small fluctuations on the 0.1 kJ/mol scale. Except for the DZ basis with the loosest threshold, the average (maximum) errors relative to the parent CCSD(F12*)(T+) method do not exceed 1 kJ/mol (1 kcal/mol). Surprisingly, there is no remarkable difference in the error measures for the atomization and reaction energies, but it is again true that the accuracy is weaker for second-row systems (see SI). Examining the errors with respect to CBS-limit CCSD(T), the results suggest that the NAB approach less benefits from error cancellation than the previous approximations. Nevertheless, the 0.3 a.u. threshold seems to be sufficient for the DZ and TZ bases, whereas for QZ, even 0.5 a.u. is adequate. Using these parameters, 55, 66, and 59% of the NAB is retained, respectively, with the DZ, TZ, and QZ basis sets (see SI). Taking into consideration these percentages and that the NAB approximation can only be used for the noniterative F12-dependent terms, moderate gain can be expected for the CCSD(F12*)(T+) model.

**Table 6:** Errors (in kJ/mol) of Approximate CCSD(F12*)(T+) Energy Differences Using the NAB Approach with Various Thresholds (in a.u.) with Respect to CCSD(F12*)(T+) and CBS CCSD(T) (in Parentheses) Reference Values for the KAW Test Set

		Threshold				
Basis set	Error	5 × 10^–1^	4 × 10^–1^	3 × 10^–1^	2 × 10^–1^	10^–1^
cc-pVDZ-F12	MAE	1.5 (4.1)	0.6 (3.6)	0.6 (3.7)	0.4 (3.5)	0.1 (3.4)
	RMS	2.1 (5.6)	0.8 (4.8)	0.8 (4.8)	0.5 (4.5)	0.2 (4.4)
	MAX	10.2(25.5)	3.2(20.6)	2.4(20.1)	2.8(18.5)	0.9(19.0)
cc-pVTZ-F12	MAE	0.2 (1.2)	0.2 (1.2)	0.1 (1.1)	0.1 (1.1)	0.0 (1.1)
	RMS	0.3 (1.5)	0.3 (1.5)	0.2 (1.4)	0.1 (1.3)	0.0 (1.4)
	MAX	1.4 (5.3)	1.5 (5.4)	0.6 (4.5)	0.3 (4.2)	0.0 (3.9)
cc-pVQZ-F12	MAE	0.1 (0.5)	0.0 (0.5)	0.0 (0.5)	0.0 (0.5)	0.0 (0.5)
	RMS	0.1 (0.6)	0.0 (0.6)	0.1 (0.6)	0.0 (0.7)	0.0 (0.6)
	MAX	0.5 (2.1)	0.2 (2.2)	0.2 (2.0)	0.2 (2.0)	0.0 (2.0)

### 3.5 Performance of the Combined FNO, NAF, and NAB Approximation

In Sections 3.2–3.4, we studied the various approximations and recommended thresholds for the case when only one of them is switched on. Here, we monitor the performance of the approximations considered if they are applied together and also propose default cutoff thresholds. At the determination of the latter, our idea was to maximize the expected gain in speed. To that end, we set out of the approximation from which the largest speedup is expected and then, we switched on the other approximations one by one in decreasing order of the expected efficiency selecting thresholds that retain the accuracy. That is, we started with the FNO approximation with the default thresholds determined in Section 3.2, 10^–4^, 10^–5^, and 10^–6^ for the DZ, TZ, and QZ bases, respectively. Then, we also deployed the NAF approximation during the CC iterations and at the evaluation of the perturbative triples correction using the thresholds given in Table 5 and selected the one with which the error measures calculated against the CBS-limit results worsen at most on the 0.1 kJ/mol scale. In the next step, the NAF approach was also turned on at the computation of the F12-dependent intermediates in a similar way, and finally, the same procedure was carried out for the NAB approximation. In this manner, default thresholds of 5 × 10^–2^, 5 × 10^–2^, and 10^–1^ a.u. were determined for the two types of NAF and the NAB approximations, respectively, independently of the basis set. For the sake of simplicity, our reduced-cost approach where all four approximations are employed simultaneously with the above thresholds will be referred to as the FNO-CCSD(F12*)(T+) method.

The results obtained with the combined approximations are presented in Table 7. In column “FNO”, the error statistics corresponding to the pure FNO approximation are given. In column “+NAF(CC)”, the errors are compiled for the case when in addition to the FNO approach, the NAF approximation is also used in the CC calculation. At the calculation of the results displayed in column “+NAF(F12)”, the NAF approximation was employed for the F12-dependent intermediates as well, while in the case of the rightmost column, all four approximations were switched on. If the errors relative to the same-basis CCSD(F12*)(T+) results are inspected, the error measures somewhat grow with the DZ basis when switching on the various approximations, but the increase is considerably lower than the intrinsic error of the method with this basis set. Using the two larger bases, the error measures hardly change excepting perhaps the MAX with the QZ basis. Considering the errors with respect to the CBS-limit, they do again practically not change, only the MAX value decreases noticeably for the DZ basis set thanks to fortuitous error cancellation. In the end, our combined approximation preserves the accuracy of the parent CCSD(F12*)(T+) model (cf. parenthesized numbers in the last column of Table 7 and Table 2).

**Table 7:** Errors (in kJ/mol) of Approximate CCSD(F12*)(T+) Energy Differences Using the Cost-Reduction Techniques Developed with the Default Thresholds with Respect to CCSD(F12*)(T+) and CBS CCSD(T) (in Parentheses) Reference Values for the KAW Test Set^a^

		Approximation			
Basis set	Error	FNO	+NAF(CC)	+NAF(F12)	+NAB
cc-pVDZ-F12	MAE	1.4 (3.3)	2.3 (3.3)	2.3 (3.3)	2.6 (3.5)
	RMS	2.2 (4.2)	3.1 (4.4)	3.1 (4.4)	3.4 (4.6)
	MAX	7.7(16.8)	9.2(17.3)	9.4(17.2)	11.1(18.1)
cc-pVTZ-F12	MAE	1.0 (1.0)	1.1 (1.0)	1.0 (1.0)	1.0 (1.0)
	RMS	1.4 (1.4)	1.6 (1.3)	1.5 (1.3)	1.5 (1.3)
	MAX	4.4 (4.8)	4.6 (4.3)	4.5 (4.2)	4.5 (3.8)
cc-pVQZ-F12	MAE	0.2 (0.4)	0.2 (0.4)	0.2 (0.4)	0.2 (0.4)
	RMS	0.3 (0.6)	0.3 (0.6)	0.3 (0.6)	0.3 (0.6)
	MAX	1.9 (2.0)	2.4 (2.1)	2.3 (2.1)	2.3 (2.0)

a See text for explanation.

For the cross-validation of our results, further benchmark calculations were performed for an independent test set, the AW set. This test set includes larger molecules, thus the CBS-limit CCSD(T) references are not available, and we can only compare the canonical and reduced-cost CCSD(F12*)(T+) results computed with the same basis set. The error statistics are presented in Table 8. As we can see, the error measures are considerably lower than the corresponding ones for the KAW test set (see numbers without parentheses in the last column of Table 7). Of course, we should realize that the KAW set includes atomization energies and reaction energies involving open-shell species, while the AW compilation is just based on reactions of closed-shell molecules. If the error measures of the closed-shell reaction energy subset of the KAW test set (see SI) are compared to those for AW, one finds that the errors are still significantly lower for the AW set with the DZ basis, while they are comparable for the two other basis sets. Thus, we have a good reason to believe that our FNO-CCSD(F12*)(T+) approach preserves its accuracy for other systems as well.

**Table 8:** Errors (in kJ/mol) of FNO-CCSD(F12*)(T+) Reaction Energies with Respect to Canonical CCSD(F12*)(T+) Reference Values for the AW Test Set

	Basis set		
Error measure	cc-pVDZ-F12	cc-pVTZ-F12	cc-pVQZ-F12
MAE	0.8	0.2	0.1
RMS	1.1	0.2	0.1
MAX	3.8	0.8	0.3

Concerning the reduction in the number of orbitals (see SI), the percentage of the dropped orbitals decreases with growing basis sets size, that is, the gain will be higher with smaller bases. Depending on the basis set, 60–90% of the NOs are retained, thus, up to 8-fold speedups are expected in the CC calculations just due to the FNO approximation. Remarkable is the large fraction of the dropped NAFs, 60–80%, which further reduces the computation time considerably if the required four-center integrals are reconstructed on-the-fly in the CC iterations. The number of the retained NAFs is significantly smaller than that for conventional CCSD(T) calculations,^18^ but of course, this is the consequence of the fact that larger fitting bases are employed for explicitly correlated CC. The FNO approximation also reduces the costs of the evaluation of the F12-dependent intermediates, but there, the NAF approximation is less efficient, and the percentage of the retained NAB functions is also relatively high. Hence, more moderate speedups are foreseen for the latter operation. The factual speedups will be presented in Section 3.6.

We note that the default truncation thresholds have been determined for the combinations of AO and auxiliary basis sets employed in our study (see Section 3.1). These are the basis set combinations that are recommended by the developers of explicitly correlated methods, but, of course, other bases are also applied in explicitly correlated CC calculations. It is very likely that our thresholds can safely be used with similar bases, for instance, if the aug-cc-pV*X*Z basis sets are chosen instead of cc-pV*X*Z-F12. In the case of less similar bases, it is recommended to run test calculations or use tighter thresholds, e.g., 10^–6^, for the FNOs. Similar holds for the choice of the explicitly correlated CC approximation. Here, we have considered the CCSD(F12*) model, but it is probable that our approximations with the cutoff parameters determined are also applicable to similar methods, such as CCSD-F12a, CCSD-F12b, or . For the approaches that treat the F12-dependent terms iteratively, a careful reconsideration of the thresholds is recommended.

### 3.6 Timings

To demonstrate the efficiency of our approximations, first, we measured the wall-clock times required for the evaluation of the F12-dependent intermediates and the CC calculations for the molecules of the AW test set and calculated the speedups. Only those 16 systems were considered that consist of at least 10 atoms because these are large enough with all three basis sets that the uncertainty of the wall time measurement does not influence our conclusions. The resulting minimal, maximal, and average speedup factors are collected in Table 9.

**Table 9:** Speedups for FNO-CCSD(F12*)(T+) Calculations against Canonical CCSD(F12*)(T+) for 16 Molecules of the AW Test Set

	Basis set		
	cc-pVDZ-F12	cc-pVTZ-F12	cc-pVQZ-F12
F12-dependent intermediates			
Minimum	3.1	2.4	1.8
Average	3.6	2.6	2.1
Maximum	4.5	2.9	2.3
CC iterations + perturbative triples			
Minimum	7.3	5.2	2.6
Average	9.1	6.0	3.6
Maximum	11.4	7.3	4.1

As can be seen, significant speedups can be achieved in all three basis sets for the CC calculations. The speedups decrease with growing basis set size, which is a consequence of the tightening FNO truncation threshold, but the gain is still remarkable even with the QZ basis. The scatter of the speedup factors is relatively low, which suggests that the approach is quite robust, and considerable savings in the computation time can be expected for any system. The tendencies are similar for the F12-dependent constant terms, but the speedup factors are roughly the half of those obtained for the CC runs. This is not surprising because, as noted above, the evaluation of the F12-dependent intermediates also scales as the size of the complementary basis, which is less efficiently reduced than that of the virtual MO basis. Moreover, significantly more NAFs are retained for the F12-dependent terms than for the Coulomb integrals used in the CC calculations. Taking into account that the CC calculations are typically 5- to 7-times more costly than the computation of the F12-dependent terms, overall speedups of 7, 5, and 3 can be expected for the DZ, TZ, and QZ bases, respectively.

### 3.7 Large-Scale Applications

Finally, we illustrate the capabilities of the presented FNO-CCSD(F12*)(T+) approach on three chemical reactions, which would otherwise be out of the reach of conventional explicitly correlated CCSD(T) codes, at least with TZ-level basis sets. The required computational resources were kept in a widely accessible range, i.e., mostly 28 CPU cores and compute times of a few days were sufficient. Thus, the presented applications should be routinely accessible for a broad audience as more and more computer clusters contain even single compute nodes with dozens of cores, while multinode parallelization is also available for the CCSD iteration and (T) parts.^57^

**Figure 1:**
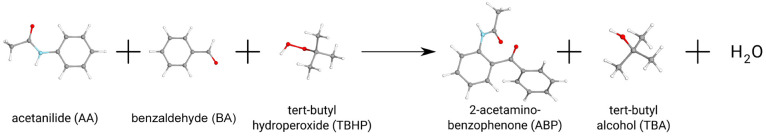
Palladium catalyzed C–H bond activation leading to the 2-acetaminobenzophenon product of 31 atoms.^97^

**Figure 2:**
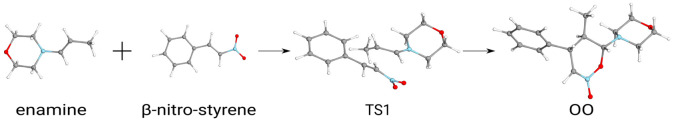
Transition state (TS1) and an intermediate (OO) of 40 atoms formed in a model organocatalytic Michael-addition reaction.^98^

**Figure 3:**
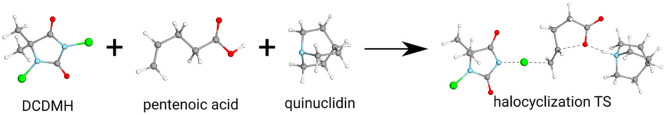
Transition state of a halocyclization reaction containing 53 atoms.^99,100^

The three applications include a palladium catalyzed C–H activation reaction,^97^ an organocatalytic Michael-addition reaction,^98^ and a halocyclization reaction^99,100^ as shown in Figures 1–3, respectively. In the first reaction, the Pd compound catalyzes the cross-dehydrogenative coupling between anilides and aromatic aldehydes forming 2-acetaminobenzophenon (ABP), as shown in Figure 1. Here, our CCSD(T)/def2-QZVPPD results for the 31-atom ABP product^57^ represents the largest molecule in the literature with QZ-level basis set in a conventional CCSD(T) computation, so we also have a def2-(T,Q)ZVPPD level extrapolated estimate for the CBS limit of the reaction energy up to this system size.

The second example is a model for an organocatalytic Michael-addition reaction^98^ with propanal and β-nitrostyrene reactants. The investigated step is the formation of a dihydrooxazine *N*-oxide (OO) intermediate from β-nitrostyrene and an enamine intermediate through a transition state (TS) denoted as TS1 (see Figure 2).^98^ The overall stereochemistry and the reaction rate of these reactions are governed by delicate interactions between the reactants and the catalyst, while the addition of the two 20-atom molecules forming the 40-atom TS1 and the OO intermediate is particularly sensitive to the basis set superposition error (BSSE). Thus, almost complete basis set convergence is required for the reliable characterization of the reaction mechanism. However, conventional CCSD(T)/QZ would be out of reach, even with our highly optimized code, just CCSD(T)/def2-TZVPPD results are available.^57^

The third example (see Figure 3) pushes the system size up to 53 atoms. Here, a halolactonization reaction similar to the model reaction of ref (99) is considered (a phenyl group is removed compared to the case of ref (99)). The barrier height is computed for the TS formed from the 1,3-dichloro-5,5-dimethylhydantoin (DCDMH) halogen source, the pentenoic acid reactant, and the quinuclidine model catalyst. Due to the incorporation of the catalyst, a commonly occurring, complicated scenario is covered where the accurate modeling of the noncovalent TS terner is hindered by considerable BSSE and the simultaneous formation or breaking of six covalent bonds.

The largest systems of the three reactions, respectively, include 657, 780, and 1041 AOs in the cc-pVDZ-F12 basis set (see SI for all basis set dimensions), which would reach the bottlenecks of frequently employed CCSD(T) programs storing the four-center integral list of multiple terabytes. Using our integral-direct code, we can still compute the CCSD(F12*)(T+)/cc-pVDZ-F12 references without approximations. Going one step further to the cc-pVTZ-F12 basis set, the 31-, 40-, and 53-atom species involve 1188, 1420, and 1882 AOs, respectively. At this scale, the largest reference computation would require extreme computational cost, so here, only the FNO-CCSD(F12*)(T+)/cc-pVTZ-F12 calculations are routinely affordable.

Considering the efficiency gain from our approximations first, the compression rates for the various MO and auxiliary basis sets are found quite homogeneous for the ABP, OO, TS1, and the halocyclization TS structures (see SI). Namely, the FNO, NAF(F12), NAF(CC), and NAB compressions are about 61%, 66%, 20%, and 79% with cc-pVDZ-F12, and 75%, 76%, 30%, and 88% with cc-pVTZ-F12, respectively. The corresponding combined FNO-NAF-NAB error in the CCSD(F12*)(T+)/cc-pVDZ-F12 correlation energies are only 0.03%–0.07% (see SI). The corresponding reaction energies and barrier heights are collected in Table 10. First, the accuracy of the cc-pVDZ-F12 energy differences is outstanding, the FNO error is 0.1 kcal/mol or lower for the ABP and OO reactions and the TS1 barrier. Additionally, the 0.6 kcal/mol (2.5 kJ/mol) deviation for the largest halocyclization barrier is well within both chemical accuracy and the MAX error of 3.8 kJ/mol reported for the AW set above in Table 8.

**Table 10:** Comparison of Reaction Energies and Barrier Heights (in kcal/mol) with and without FNO Approximations as well as with and without Explicit Correlation

		CCSD(T)	CCSD(F12*)(T+)	
Reaction	Approximation	def2-XZVPPD^a^	cc-pVDZ-F12	cc-pVTZ-F12
ABP	none	–73.82^b^	–73.76	
	FNO^c^	–73.82	–73.65	–73.25
OO	none	–23.75	–21.35	
	FNO^c^	–23.66	–21.33	–21.52
TS1	none	4.89	7.38	
	FNO^c^	5.01	7.42	7.59
Haloc. TS	none		9.15	
	FNO		9.78	9.30

a def2-QZVPPD for ABP and def2-TZVPPD for OO and TS1.

b The def2-(T,Q)ZVPPD CBS extrapolated value is −73.50 kcal/mol.

c Tighter FNO threshold of 10^–5^ is used for FNO-CCSD(T).^18^

Regarding the level of basis set convergence, our CCSD(T)/def2-(T,Q)ZVPPD ABP reaction energy of −73.50 kcal/mol compares excellently to the −73.65 and −73.25 kcal/mol results obtained with FNO-CCSD(F12*)(T+) and the cc-pVDZ-F12 and cc-pVTZ-F12 basis sets, respectively. As noted above, the CCSD(T)/def2-TZVPPD results for the OO and TS1 reaction energy and barrier are still affected by a notable BSSE, namely, more than 2 kcal/mol deviation is found when the CCSD(T)/def2-TZVPPD results are compared to those obtained with CCSD(F12*)(T+). On the other hand, the 0.02–0.04 kcal/mol FNO error with the cc-pVDZ-F12 basis set and the sub-0.2 kcal/mol deviation of the cc-pVDZ-F12 and cc-pVTZ-F12 results for OO and TS1 indicate excellent convergence in terms of both the FNO approximation and the basis set size used for CCSD(F12*)(T+). Finally, a somewhat higher difference of 0.5 kcal/mol found for the halocyclization barrier between the cc-pVDZ-F12 and cc-pVTZ-F12 level FNO-CCSD(F12*)(T+), which is still satisfactory considering the size and complexity of the system.

For these cases, our approximations do not affect the accuracy of FNO-CCSD(F12*)(T+) compared to the level of the basis set convergence, and probably in such cases, it is worthwhile experimenting with the relaxation of the tight FNO and other thresholds used here depending on the target accuracy. Additionally, at least for these three cases, even the FNO-CCSD(F12*)(T+)/cc-pVDZ-F12 results are found within 0.2–0.5 kcal/mol of the best available, presumably converged results, which could be a satisfactory level of accuracy for most chemical applications.

Finally, let us analyze the wall times corresponding to the above conventional and FNO-accelerated CCSD(F12*)(T+) computations (see Table 11). Remarkably, the about 0.6-, 1.6-, and 8.6-day long CCSD(F12*)(T+)/cc-pVDZ-F12 computations can be sped up by a consistent factor of about 5. Consequently, the FNO-CCSD(F12*)(T+)/cc-pVDZ-F12 jobs were completed only within 2.5, 7.5, and 44 h on 28 cores. Considering the excellent accuracy found above, the FNO-CCSD(F12*)(T+)/cc-pVDZ-F12 combination running only a few days on a few dozen cores provides a routinely affordable reference method on most computer hardware up to at least 50 atoms. Compared to the 68-h (224 cores) performance of DF-CCSD(T)/def2-QZVPPD for the ABP molecule, the 2.5-h runtime (28 cores) of FNO-CCSD(F12*)(T+)/cc-pVDZ-F12 represents a drastic improvement of 2 orders of magnitude at practically the same level of basis set convergence. Similarly, the considerably less converged DF-CCSD(T)/def2-TZVPPD results took 32 h (112 cores) for the OO and TS1 systems, which is an order of magnitude more expensive than the present FNO-CCSD(F12*)(T+)/cc-pVDZ-F12 jobs taking only 7.5 h (28 cores). Compared to their cc-pVDZ-F12 counterparts, the 31-, 40-, and 53-atom FNO-CCSD(F12*)(T+)/cc-pVTZ-F12 computations are about a factor of 10–16 times more demanding, taking about 1.6, 3.8, and 19.8 days of wall time (with 28, 56, and 84 cores, respectively). Thus, the combined FNO-NAF-NAB methodology also brings down the costs of CCSD(F12*)(T+)/cc-pVTZ-F12 level computations to a routinely affordable, few-day compute time for at least up to 40 atoms. Somewhat larger 50+ atom computations are still feasible using more cores with larger CPU time investments.

**Table 11:** Wall Times (in Minutes) Separately for the Construction of the F12-Dependent Intermediates and the Combined CCSD(F12*) Iterations and (T+) Correction Steps with and without FNO Approximations^a^

				CCSD(F12*)(T+)		FNO-CCSD(F12*)(T+)		
Species	Atoms	Basis set	AOs	F12	CC	F12	CC	Speedup
ABP	31	cc-pVDZ-F12^b^	657	66	728	25	123	5.4
		cc-pVTZ-F12^b^	1188	-	-	194	2177	-
OO and TS1	40	cc-pVDZ-F12^b^	780	125	2110	55	395	5.0
		cc-pVTZ-F12^c^	1420	-	-	541	4864	-
Haloc. TS	53	cc-pVDZ-F12^b^	1041	435	11879	199	2485	4.6
		cc-pVTZ-F12^d^	1882	-	-	1381	27173	-

a The last column reports the overall speedups gained via the FNO approximations.

b 28 cores.

c 56 cores.

d 28 cores for the F12 intermediates, 84 cores for CCSD(T).

The minimal memory and disk space requirements for the most demanding steps of the CCSD(F12*)(T+) calculations are collected in the SI for the largest computations. These expressions are evaluated according to the storage requirement of our codes detailed in ref (57), while in practice, at least about twice as much memory was allocated to decrease the amount of repeated integral evaluations in our integral-direct algorithms. Compared to the molecule sizes of 31–53 atoms, we find the about 5–25 GB and 44–203 GB minimal memory requirements with the cc-pVDZ-F12 and cc-pVTZ-F12 basis sets, respectively, highly accessible. The approximately 4–7 times as much disk requirement needed mostly for the storage of the cluster amplitudes and error vectors is similarly accessible.

## 4 Conclusions

Several possibilities have been explored for the reduction of the computational expenses of explicitly correlated CCSD(T) methods. The FNO and NAF techniques, which were previously employed for conventional CCSD(T), have been adapted to explicitly correlated CCSD(T) to decrease the size of the virtual MO and density fitting bases, respectively. In addition, a new approximation, termed the NAB approach, has been proposed to reduce the size of the complementary auxiliary basis. Our results show that the FNO approach is as efficient as for conventional CCSD(T) and contributes to the largest extent to the speedups observed. The NAF approximation is extremely advantageous for reducing the size of the auxiliary basis required for the fitting of Coulomb integrals during the CC calculations. It is less efficient for the calculation of the F12-dependent constant terms, where other types of integrals are also approximated by DF. The NAB scheme only moderately reduces the complementary basis, but it is anticipated that this approximation will be rather useful for explicitly correlated local correlation models, where the treatment of the corresponding three-center integrals is a bottleneck. The efficiency of the combined FNO-NAF-NAB approach decreases with increasing basis set size, but 3-fold speedups can be expected even with quadruple-ζ bases. At the same time, the errors of energy differences with respect to the corresponding CBS-limit values are virtually not changed. Our results also demonstrate that the combined cost-reduction approach considerably extends the reach of explicitly correlated CCSD(T). Namely, the basis set limit of CCSD(T) can now be routinely approached well within sub-kcal/mol accuracy for molecules of up to 50 atoms with widely affordable, moderate computational resources and few-day computation times.

## Supporting Information Available

Calculated correlation, atomization, and reaction energies; number of retained orbitals; and the measured computation times (XLSX)
